# Indirect treatment comparison of lanadelumab and a C1-esterase inhibitor in pediatric patients with hereditary angioedema

**DOI:** 10.57264/cer-2024-0110

**Published:** 2025-01-21

**Authors:** Maureen Watt, Rachel Goldgrub, Mia Malmenäs, Katrin Haeussler

**Affiliations:** 1 Takeda Development Center Americas, Inc., Lexington, MA 02421, USA; 2 ICON plc, Insights, Evidence & Value – Health Economics & Epidemiology, Vancouver, British Columbia, Canada; 3 ICON plc, Insights, Evidence & Value – Health Economics & Epidemiology, Stockholm, Sweden; 4 ICON plc, Insights, Evidence & Value – Health Economics & Epidemiology, Langen, Germany

**Keywords:** C1 esterase inhibitor, comparative effectiveness, hereditary angioedema, indirect treatment comparison, lanadelumab, prophylaxis

## Abstract

**Aim:**

To compare the efficacy and safety of lanadelumab versus other approved long-term prophylaxis (LTP) treatments in patients with pediatric hereditary angioedema (HAE) aged <12 years.

**Materials & methods:**

A systematic literature review was conducted to identify studies of LTP in patients with HAE aged <12 years. Two studies met the inclusion criteria in an indirect treatment comparison of efficacy and safety data in pediatric HAE patients. These were for lanadelumab (SPRING, NCT04070326) and intravenous-C1-esterase inhibitor (C1-INH[IV], NCT02052141). A propensity score analysis used individual patient-level data from both studies in a logistic regression model to estimate inverse probability weights. To avoid convergence issues and an underpowered analysis due to the small sample size (n = 29), the base case was defined as Poisson regression analyses on monthly attack rate adjusting for one covariate (baseline attack rate). Model selection among unadjusted, adjusted and weighted regression models was conducted through the Akaike and Bayesian Information Criteria.

**Results:**

Lanadelumab 150 mg every 2 weeks (Q2W) reduced the monthly HAE attack rate by 82.1% versus C1-INH(IV) 1000 IU twice weekly (every 3–4 days [BIW]; rate ratio [RR], 0.1792 [95% CI: 0.0296–1.0853]) and by 88.9% versus C1-INH(IV) 500 IU BIW (RR: 0.1107 [95% CI: 0.0234–0.5239]). Treatment with lanadelumab Q2W reduced the risk of total adverse events by 56.2% versus C1-INH(IV) 1000 IU BIW (RR:0.4377 [95% CI: 0.1536–1.2469]) and by 66.0% versus C1-INH(IV) 500 IU BIW (RR: 0.3401 [95% CI: 0.1234–0.9371]).

**Conclusion:**

This exploratory analysis suggested a trend toward greater efficacy and fewer adverse events with lanadelumab 150 mg Q2W compared with C1-INH(IV) BIW 1000 IU and 500 IU in pediatric patients with HAE. Future studies could potentially assess larger samples over longer periods of time for the long-term preventative efficacy, safety and tolerability of lanadelumab and C1-INH(IV).

## Graphical abstract

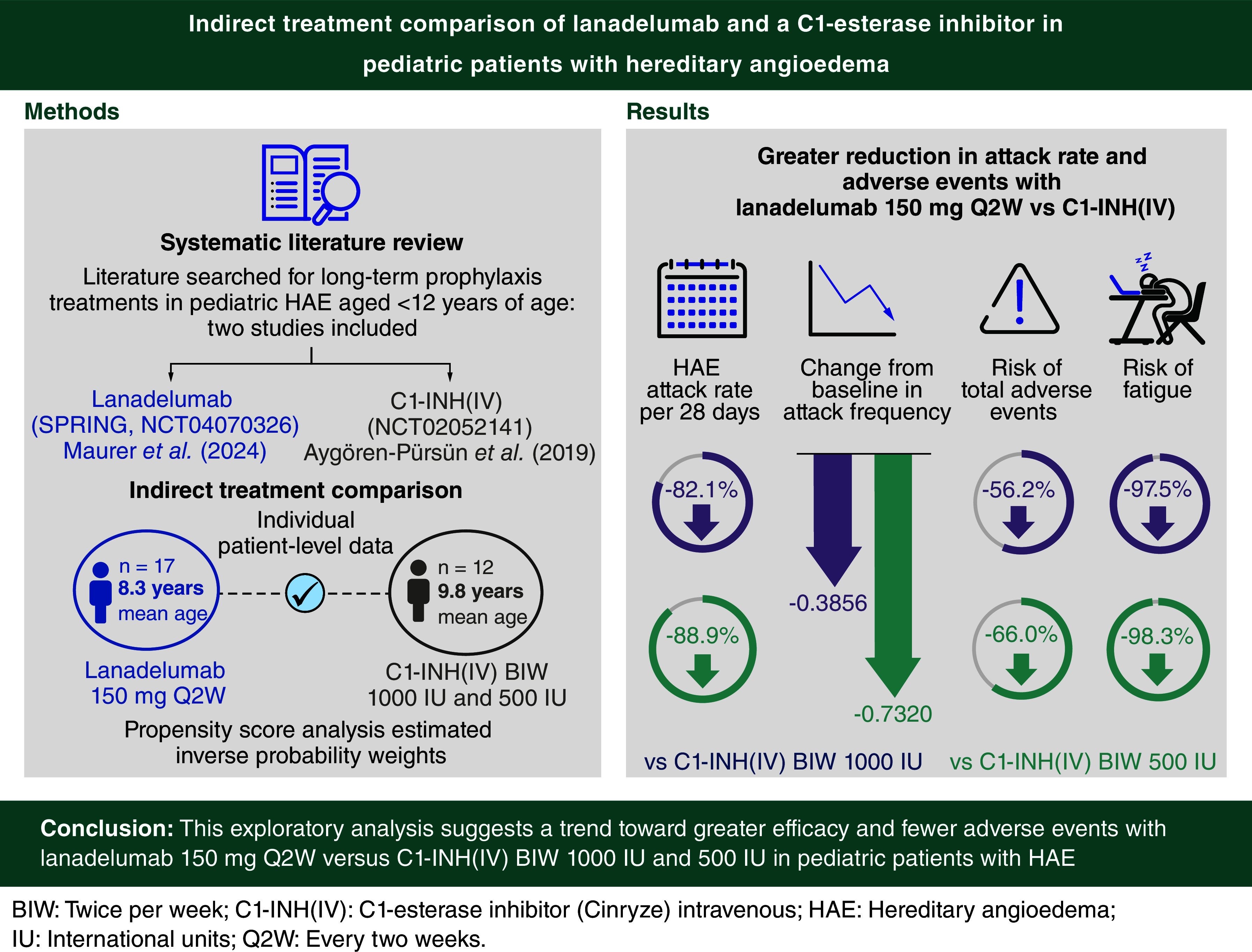

Hereditary angioedema (HAE) is a rare, genetic disease characterized by episodes of skin and mucous membrane swelling involving the upper airway and GI tract [1]. HAE attacks can be painful and disfiguring, and laryngeal attacks carry a risk of fatal asphyxiation [2–4]. Most HAE cases are caused by mutations in the SERPING1 gene, which encodes for the C1-esterase inhibitor (C1-INH) [2]. These mutations result in either a plasma deficiency (HAE Type I) or dysfunction (HAE Type I) of C1-INH, with children having a 50% chance of inheriting HAE from an affected parent [5,6]. Symptoms can manifest at any age, but usually appear during childhood, worsen during puberty and persist throughout life [2–4,7,8]. The typical age of onset is 6–11 years and can occur as early as infancy [9–12], with an earlier onset predicting a more severe course of the disease [2,4,9,10].

HAE therapy includes acute (on-demand), short-term prophylaxis and long-term prophylaxis (LTP), with international clinical guidelines considering pediatric patients <12 years of age as children and 12–17 years of age as adolescents [4,13]. The use of intravenous C1-INH or icatibant is recommended for the on-demand treatment of HAE attacks in pediatric patients (<12 years of age). When these therapies are not available, the guidelines recommend treating attacks in pediatric patients with solvent/detergent-treated plasma (SDP) or, in the absence of SDP, fresh frozen plasma [4]. For LTP, the use of a plasma-derived C1-INH, berotralstat or lanadelumab is recommended in adults and adolescents [4]. The only approved therapy for LTP in patients with HAE aged 2 years and older is lanadelumab (Takhzyro, Takeda) [14,15], which was approved by the US FDA and European Medicines Agency for this age group before publication of the most recent clinical guidelines [4]. Intravenous-C1-INH [C1-INH(IV)], Cinryze, Takeda), which is a human plasma-derived C1-INH [16,17], and subcutaneous-C1-INH [C1-INH(SC)], Haegarda and Berinert 2000/3000, CSL Behring) [18,19] are approved for LTP in patients 6 years and older.

Living with HAE can be difficult for patients and their families, both during and between swelling episodes [20]. The aim of LTP is to achieve full control of the disease and to normalize patients' lives [4]. Therefore, it is important for effective LTP options to be available for pediatric patients with HAE. The following treatments have been investigated as LTP in children: C1-INH(IV), lanadelumab and C1-INH(SC) [21–23].

The effectiveness and tolerability of LTP with C1-INH(IV) in children aged 6–11 years (n = 12) was demonstrated in a randomized, phase III, single-blind, crossover study as reported by Aygören-Pürsün *et al.* [21] (hereafter referred to as the C1-INH study). Following a 12-week baseline period, patients received 500 or 1000 IU C1-INH(IV) twice weekly (every 3–4 days [BIW]) for 12 weeks before switching to the alternate dose for 12 weeks. The mean (standard deviation [SD]) within-patient difference (-0.4 [0.58]) for monthly number of angioedema attacks with both doses was significant (p = 0.035 [90% CI, -0.706 to -0.102]), with no serious adverse events or discontinuations reported [21].

The efficacy and safety of lanadelumab 150 mg every 2 weeks (Q2W) or every 4 weeks (Q4W) were evaluated for the prevention of HAE attacks in patients aged 2 to <12 years in the phase III, open-label, multicenter SPRING study (n = 21) [23]. This trial enrolled 21 patients with HAE type I/II and at least one investigator-confirmed HAE attack during baseline. Over 52 weeks of treatment, patients aged 2 to <6 years received lanadelumab 150 mg Q4W and patients aged 6 to <12 years received 150 mg lanadelumab Q2W. Patients who received Q2W dosing and were well controlled at 26 weeks (study treatment period A) could switch to Q4W in study treatment period B in the trial. The attack rate was reduced by 94.8% from baseline over 52 weeks of treatment, with 76.2% of patients remaining attack-free during the full follow-up period [23].

The efficacy and safety of C1-INH(SC) in patients with HAE have been demonstrated in an open-label extension (OLE) of the pivotal phase III (COMPACT) study of C1-INH(SC) [22]. Patients were eligible for inclusion if they were aged 6 years or older, but only 10 patients recruited for the study were younger than 18 years and only three were younger than 12 years [22]. Patients were treated for up to 140 weeks with C1-INH(SC) in the OLE. C1-INH(SC) was well tolerated and had a sustained prophylactic effect, with a median attack rate of 1.0 attack per year in patients treated with the C1-INH(SC) dose of 60 IU/kg [22].

To our knowledge, there are no head-to-head studies comparing LTP with lanadelumab or C1-INH for the prevention of HAE attacks in pediatric patients. The objective of this indirect treatment comparison (ITC) was to compare the efficacy and safety of lanadelumab versus other approved LTP treatments in pediatric HAE (<12 years of age).

## Methods

### Systematic literature review

An initial search was run in December 2016 to capture randomized controlled trials (RCTs) and non-randomized prospective interventional studies of LTP according to the criteria in Supplementary Table 1. As the phase III SPRING study did not contain a placebo arm, any ITC would not be linked by a common treatment comparison in a matching-adjusted indirect comparison (MAIC). Therefore, an updated search was conducted in February 2021 to include single-arm observational studies as well as to capture newly available HAE treatments. Additional searches occurred in March 2021 and in July 2022 to update information from congresses, with the July 2022 search including results from the American Academy of Allergy, Asthma and Immunology (AAAAI) conference. In the final search in October 2022, all previously identified studies were reviewed to identify studies that reported data on pediatric patients aged <12 years; in addition, conference abstracts were updated. Proceedings from the following conferences were searched for in 2018, 2019 and 2020: (1) American Association of Immunologists, (2) American College of Allergy, Asthma and Immunology, (3) European Academy of Allergy and Clinical Immunology and (4) International Society for Pharmacoeconomics and Outcomes Research. In the July 2022 search, the AAAAI conference was reviewed from 2015 to 2020. In the October 2022 update, all five conferences were searched for abstracts from 2021 and 2022 (if available). Two trial databases were also searched to identify ongoing trials: ClinicalTrials.gov and the WHO International Clinical Trials Registry Platform Search Portal.

Searches of MEDLINE, Embase and Cochrane databases as well as congress proceedings were run in accordance with the Preferred Reporting Items for Systematic Reviews and Meta-Analyses (PRISMA) statement, the Centre for Reviews and Dissemination and the Cochrane Collaboration [24–26]. Titles, abstracts and full texts were reviewed in a double-blind manner by two systematic reviewers. A risk of bias assessment was conducted based on recommendations from the National Institute for Health and Care Excellence [27]; it did not include observational studies, non-RCTs, or conference abstracts. A PRISMA diagram outlining the inclusion and exclusion of studies throughout the review process is presented in Supplementary Figure 1.

### Indirect treatment comparison

#### Outcomes

The safety and efficacy outcomes reported in the SPRING lanadelumab study [23] were considered, since the objective of this ITC was to compare lanadelumab's effectiveness against other approved LTP treatments in pediatric HAE (<12 years).

#### Prognostic variables & baseline characteristics

Adjustment for potential imbalances in relevant prognostic variables between cohorts was conducted through inverse probability of treatment weighting (IPTW; Supplementary methods). Based on the availability and data quality of the variables in the SPRING and the C1-INH studies, age, sex, baseline HAE attack rate, body mass index and race were chosen as the prognostic variables relevant for unanchored comparison. Baseline and prognostic factors of the SPRING and the C1-INH studies per individual patient data are shown in Supplementary Table 2.

#### Statistical analysis

Differences in baseline characteristics between the two cohorts were evaluated using *t*-tests for continuous variables and chi-square tests for categorical outcomes. The HAE attack rate as well as the safety outcomes calculated from the Poisson regression model were reported as rate ratios (RRs) with 95% confidence intervals (CIs). For continuous outcomes (difference in change from baseline in HAE attacks), mean differences with 95% CIs were estimated through linear regression and p-values were reported. All summaries and analyses were performed using SAS^®^ version 9.4 or higher.

#### Propensity score model

The propensity score analysis was performed using a base propensity score model. To avoid convergence issues and an underpowered analysis due to the small sample size, the base case was defined as Poisson regression analyses on monthly attack rate adjusting for exclusively one covariate (baseline attack rate). Model selection among unadjusted, adjusted and weighted regression models was conducted through the Akaike information criterion (AIC) and Bayesian information criterion (BIC); the lowest AIC and BIC values indicate the best model fit to the data. The overlap of the distribution of the propensity scores across the SPRING trial cohort [23] and the C1-INH study control cohort [21] was assessed by examining a histogram distribution graph of propensity scores across cohorts. Covariate-adjusted comparisons of HAE rates and binary safety outcomes between cohorts were performed using IPTW. After IPTW, the covariate in the base model became similar in the two cohorts, and the standardized difference on baseline HAE attack rate was reduced from -0.67 to -0.07 (Supplementary Table 3).

Additional analyses included the covariates baseline attack rate, age and sex (Model 2) or baseline attack rate, age, sex, race and body mass index (Model 3). However, the histogram of propensity scores for Models 2 and 3 had a poorer overlap between the two cohorts and a decision was made to focus on the results of the base model. No analyses could be conducted for the comparison of lanadelumab 150 mg Q4W due to a small sample size (n = 4).

## Results

### Systematic literature review

The systematic literature review (SLR) was conducted to identify studies of LTP in patients with HAE aged <12 years for inclusion in the ITC. The initial searches based on criteria in Supplementary Table 1 yielded 59 studies reported in 102 publications. The 59 studies were RCTs, prospective non-randomized interventional studies, or observational studies evaluating treatments for HAE in patients of any age with HAE and a mixed population of age groups. After excluding studies of patients with HAE either aged ≥18 years or a mixed population of age groups, there were 15 studies reported in 19 publications of pediatric patients or subgroups of patients aged 2 to 17 years (Busse *et al.* [28] was counted twice as it reported on HELP and HELP OLE) [7,21,28–43]. The HAE clinical guidelines consider pediatric patients as children aged <12 years [4,13], and this was the age group in the phase III SPRING study [23]. Therefore, studies that did not include patients aged <12 years were excluded. The COMPACT OLE of C1-INH(SC) was also excluded as it included only three patients with HAE <12 years of age and insufficient baseline information to conduct an MAIC with C1-INH(SC) in pediatric patients aged <12 years [44]. As a result, 17 publications of patients aged ≥12 years and <18 years were excluded. Thus, two studies in three publications of patients aged <12 years receiving LTP for HAE met the inclusion criteria and were used for the MAIC. These were the SPRING study for lanadelumab (the full-text publication for this trial was added in *post hoc*) [23,40] and the 2019 C1-INH study [21] (further details in Supplementary Figure 1).

### Indirect treatment comparison

Given that the SLR identified only two relevant studies and that the study team had access to patient-level data from both, it was determined that an ITC using individual patient data was the most appropriate methodology. The lanadelumab treatment arm for the ITC was derived from the SPRING study [23]. The C1-INH(IV) comparator arm was from the C1-INH study [21]. An overview of the design of the SPRING trial [23] and the C1-INH study [21] is shown in Supplementary Table 4. The ITC included only SPRING study patients who received Q2W dosing (n = 17), with outcomes reported up to week 26 (study period A). SPRING study patients who received Q4W dosing in period A were <6 years of age, and thus would not be comparable to the older patient group (6–11 years) of the C1-INH study [21]. Additionally, outcomes for study period B were not included in the ITC because switching treatment arms further reduced sample sizes. The C1-INH study comprised 12 patients aged ≥6 to <12 years with a confirmed HAE type I/II diagnosis, functional CI-INH level <50% of normal and an average of ≥1.0 (≥2.0 in Germany) attacks/month of moderate or severe intensity or requiring acute treatment. After a 12-week observation period, patients received 500 or 1000 IU C1-INH BIW for 12 weeks before crossing over to the alternative dose for 12 weeks [21].

After identifying the two relevant studies, the following outcomes were determined to be appropriate for an MAIC: number of HAE attacks during the study period calculated per month, change from baseline in attack frequency, total adverse events and fatigue. Fatigue was included because it was reported and measured consistently between the SPRING and C1-INH studies.

#### Prognostic & baseline characteristics

The SPRING Q2W dosing cohort included 17 patients, with a mean age of 8.29 years. The C1-INH study cohort included 12 patients with a mean age of 9.75 years. Baseline characteristics are presented in Supplementary Table 2.

#### Propensity score analysis

The results of the regression base model adjusted for the propensity score had the lowest AIC and BIC and it was thus selected as the best model fit to the data.

##### HAE attack rate per 28 days

**Figure 1.**
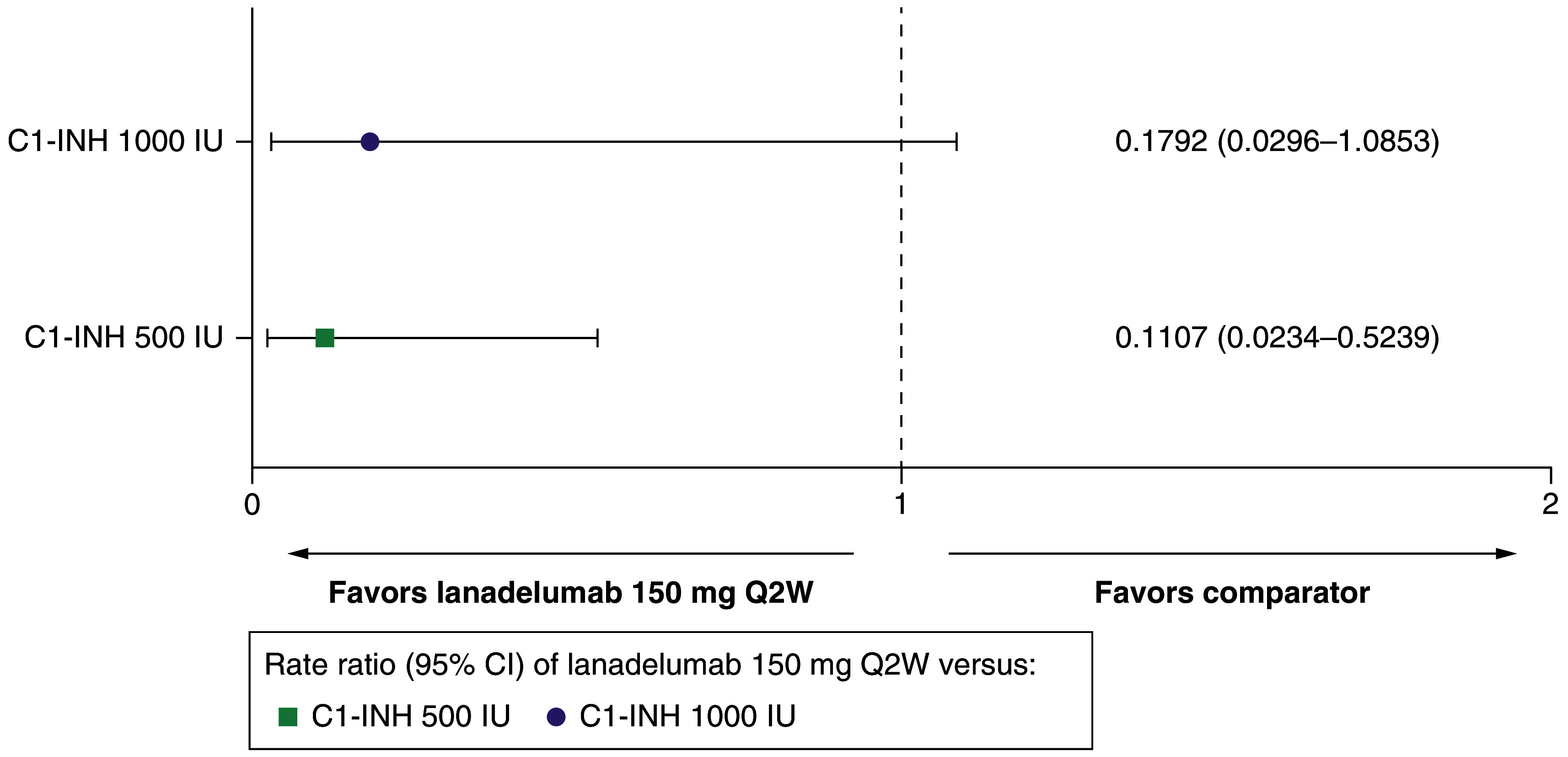
Hereditary angioedema attack rate per 28 days for lanadelumab 150 mg every 2 weeks versus C1-INH 500 IU and 1000 IU. C1-INH: C1-esterase inhibitor; CI: Confidence interval; IU: International unit; Q2W: Every 2 weeks.

Treatment with lanadelumab Q2W reduced the rate of HAE attack per 28 days by 82.1% compared with C1-INH(IV) 1000 IU (RR: 0.1792 [95% CI: 0.0296–1.0853]), and by 88.9% compared with C1-INH(IV) 500 IU (RR: 0.1107 [95% CI: 0.0234–0.5239]) (Table 1 and Figure 1).

**Table 1.** Indirect comparison of outcomes for lanadelumab 150 mg Q2W versus C1-INH 500 IU and 1000 IU.

Treatment effect	Approach	Comparator	C1-INH 1000 IU	C1-INH 500 IU
HAE attack rate per 28 days	Regression with propensity score^†^	Rate ratio (95% CI)	0.1792 (0.0296 to 1.0853)	0.1107 (0.0234 to 0.5239)
Change from baseline in attack frequency	Regression with propensity score^†^	Mean difference (95% CI)	-0.3856 (-0.9612 to 0.1900)	-0.7320 (-1.3838 to 0.0801)
Total adverse events	Weighted regression^†^	Rate ratio (95% CI)	0.4377 (0.1536 to 1.2469)	0.3401 (0.1234 to 0.9371)
Fatigue	Regression^†^	Rate ratio (95% CI)	0.0250 (0.0004 to 1.5550)	0.0170 (0.0003 to 1.0459)

† The best model fit according to AIC/BIC, selected from base models accounting for baseline attack rate (model 1).

AIC: Akaike Information Criterion; BIC: Bayesian information criterion; C1-INH: C1-esterase inhibitor; IU: International unit; Q2W: Every 2 weeks.

##### Change from baseline in attack frequency

**Figure 2.**
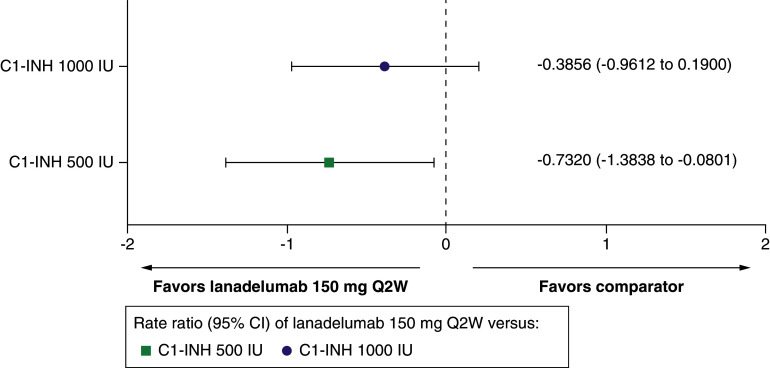
Change from baseline in attack frequency for lanadelumab 150 mg every 2 weeks versus C1-INH 500 IU and 1000 IU. C1-INH: C1-esterase inhibitor; CI: Confidence interval; IU: International unit; Q2W: Every 2 weeks.

The mean difference in change from baseline in attack frequency with lanadelumab Q2W was -0.3856 compared with C1-INH(IV) 1000 IU (95% CI: -0.9612 to 0.1900) and -0.7320 compared with C1-INH(IV) 500 IU (95% CI: -1.3838 to -0.0801) (Table 1 & Figure 2).

##### Total adverse events

**Figure 3.**
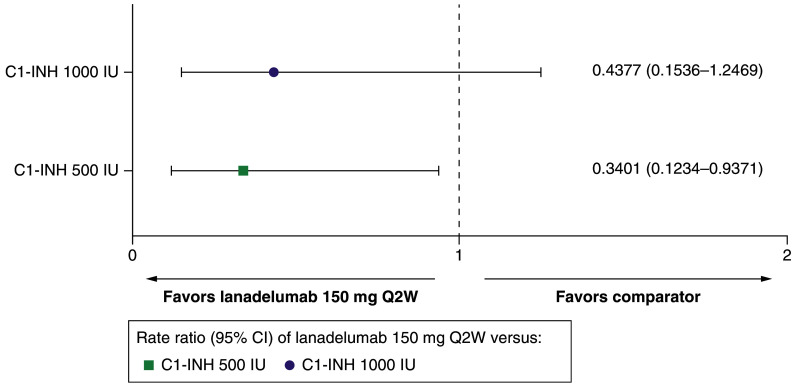
Total adverse events for lanadelumab 150 mg every 2 weeks versus C1-INH 500 IU and 1000 IU. C1-INH: C1-esterase inhibitor; CI: Confidence interval; IU: International unit; Q2W: Every 2 weeks.

Treatment with lanadelumab Q2W reduced the risk of total adverse events by 56.2% compared with C1-INH(IV) 1000 IU (RR: 0.4377 [95% CI: 0.1536–1.2469]), and by 66.0% compared with C1-INH(IV) 500 IU (RR: 0.3401 [95% CI: 0.1234–0.9371]) (Table 1 & Figure 3).

##### Fatigue

**Figure 4.**
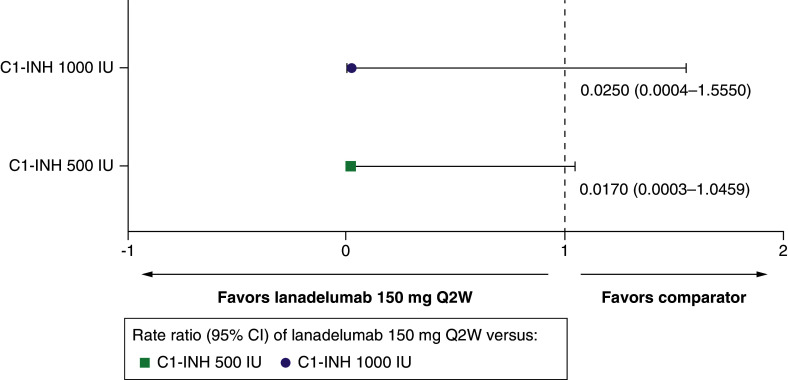
Fatigue for lanadelumab 150 mg every 2 weeks versus C1-INH 500 IU and 1000 IU. C1-INH: C1-esterase inhibitor; CI: Confidence interval; IU: International unit; Q2W: Every 2 weeks.

Treatment with lanadelumab Q2W reduced the risk of fatigue by 97.5% compared with C1-INH(IV) 1000 IU (RR: 0.0250 [95% CI: 0.0004–1.5550]), and by 98.3% compared with C1-INH(IV) 500 IU (RR: 0.0170 [95% CI: 0.0003–1.0459]) (Table 1 & Figure 4).

## Discussion

This ITC suggests a trend toward greater efficacy and fewer adverse events for LTP with lanadelumab 150 mg versus LTP with C1-INH(IV) 1000 IU and 500 IU in pediatric patients with HAE. Owing to the small sample size (n = 17 patients in the SPRING 150 mg Q2W cohort [23] and 12 patients in the C1-INH study [21]), these results should be interpreted as exploratory, with the goal of determining the direction of point estimates and identifying areas for future research. This could include designing studies with larger sample sizes. However, there are challenges in recruiting patients aged <12 years with HAE, as most patients are diagnosed with HAE in adolescence, and due to the rare nature of HAE, there is often a significant delay in diagnosis after symptom onset [45]. Of note, the prevalence of HAE in children in two European studies has been reported to be 2.51:100,000 [46] and 1:100,000 [47], which compares to an estimated prevalence of 1:50,000 in US adults [48,49]. In addition, while symptoms may begin in early childhood, HAE attacks are typically infrequent in children before puberty [50].

While there are several novel treatments for patients with HAE aged >12 years, the options for pediatric patients with HAE aged less than 12 years are limited. This could be because HAE clinical trials typically recruit only older patients, and the rare nature of HAE in this age group [46] leads to a small sample size, making it difficult to collect data and support final conclusions. The results of the SLR informed this ITC; it was expected that according to the international guidelines, C1-INH(IV) and C1-INH(SC) would be the comparators to lanadelumab in pediatric patients with HAE, as these are the only LTP options recommended in patients aged <12 years. However, trials of C1-INH(SC) provided insufficient data in patients <12 years of age, with only the C1-INH study for C1-INH(IV) [21] suitable for inclusion as a comparator. The study team had access to patient data from the SPRING and C1-INH studies, which allowed an ITC to be performed using patient level data. The use of individual patient data has the advantage over aggregate data by accounting for heterogeneity in underlying studies, thus improving statistical power [51,52].

The findings from this ITC are in line with those from an ITC of lanadelumab and C1-INH(IV) in patients with HAE aged ≥6 years [53]. Data in that study were from the 26-week, placebo-controlled HELP study of lanadelumab [54] and the 12-week, parallel-arm, crossover CHANGE trial of C1-INH(IV) [55]. Results from Bayesian and frequentist methods suggested that lanadelumab reduced HAE attack rates by 46–73% compared with C1-INH(IV) [53]. A more recent ITC also used individual patient data (n = 231) from the HELP and CHANGE trials. Lanadelumab showed a statistically significant improvement in the reduction of attack rates versus C1-INH(IV) in patients with HAE receiving LTP (monthly rate ratio: 0.486; 95% CI: 0.253–0.932) [5]. Although not assessed in the present study, lanadelumab has the advantage of subcutaneous delivery, which can be more convenient, has a shorter infusion time and provides lower systemic complications than intravenous administration (e.g., C1-INH[IV]) [14,17,44,56].

## Conclusion

This exploratory analysis suggested a trend toward greater efficacy and improved safety of LTP with lanadelumab 150 mg Q2W versus LTP with C1-INH(IV) 1000 IU and 500 IU in pediatric patients with HAE. These findings support the efficacy of lanadelumab in reducing the HAE attack rate in pediatric patients. If challenges relating to the rare nature of pediatric HAE can be addressed, future studies could assess larger samples over longer periods of time to provide more robust information on the long-term preventative efficacy, safety and tolerability of lanadelumab and C1-INH. To supplement future research, real-world evidence from natural history studies of HAE could also be used to compare outcomes in pediatric patients with HAE. In addition, the formation of regional or international multicenter collaborations could potentially increase the number of patients, expertise and study resources available for studies of pediatric HAE.

## Summary points

- Hereditary angioedema (HAE) is a rare, inherited disease characterized by recurrent episodes of swelling that are potentially fatal.
- Symptoms of HAE usually present during childhood and can occur as early as infancy.
- Long-term prophylaxis (LTP) aims to reduce the burden of HAE by preventing attacks and improving quality of life.
- There are no head-to-head studies comparing the efficacy of LTP with lanadelumab or C1-esterase inhibitor (C1-INH) for the prevention of HAE attacks in pediatric patients.
- The objective of this study was to compare the efficacy and safety of lanadelumab with other approved LTP treatments in pediatric HAE.
- A systematic literature review identified two studies that met the inclusion criteria of patients with HAE aged <12 years receiving LTP: lanadelumab (SPRING, NCT04070326) and intravenous-C1-esterase inhibitor (2019 C1-INH study, NCT02052141).
- An indirect treatment comparison was conducted with propensity score methodology using individual patient data from these studies to compare the efficacy and safety of lanadelumab 150 mg every 2 weeks (Q2W) with C1-INH(IV) BIW.
- This exploratory analysis suggested a trend toward greater efficacy and fewer adverse events with lanadelumab 150 mg Q2W versus C1-INH(IV) BIW 1000 IU and 500 IU in pediatric patients with HAE.

## Supplementary Material


