# Supplementary material for: Indirect treatment comparison of lanadelumab and a C1-esterase inhibitor in pediatric patients with hereditary angioedema
Source: J Comp Eff Res. 2025 Jan 21;14(2):e240110. doi: 10.57264/cer-2024-0110 (PMC11773901; doi:10.57264/cer-2024-0110)
Supplement: Supplementary file 1 [file cer-14-240110-s1.docx]

# Supplementary information

## Supplementary methods

**Inverse probability weighting**

Inverse probability of treatment weighting (IPTW) was performed using weights from estimated propensity scores. Weights were calculated from propensity scores so that resulting estimates refer to the average treatment effect (ATE) [1].

IPTW computes the weight for the $j^{th}$ observation with propensity score $p_{j}$ as

$$\omega_{j}=\left\{ \begin{aligned} \frac{1}{p_{j}} \text{for observations in the SPRING cohort} \\ \frac{1}{1-p_{j}} \text{for observations in the }\text{C1-INH }\text{ cohort} \end{aligned} \right.$$

Difficulties can arise when individuals are assigned very large weights, which happens when estimated propensity scores are close to zero or one. Stabilized weights were used to avoid large weights increasing the variability of the estimated treatment effect. The purpose of using stabilized weights is to reduce the weights of either those treated subjects with low propensity scores or those untreated subjects with high propensity scores. Stabilized weights are obtained by multiplying the IPTW by the marginal probability of receiving the actual treatment received, i.e.

$$\omega_{j}^{*}=\left\{ \begin{aligned} p_{t}\omega_{j}= \frac{p_{t}}{p_{j}} \text{for observations in the SPRING cohort} \\ {(1-p}_{t}) \omega_{j}= \frac{{1-p}_{t}}{1-p_{j}} \text{for observations in the }\text{C1-INH }\text{ cohort} \end{aligned} \right.$$

where $p_{t}= N_{SPRING}/ (N_{SPRING}+ N_{C1-INH})$ is the proportion of individuals in the SPRING cohort.

To assess balance in baseline characteristics between cohorts before and after weighting and matching, absolute standardized differences were calculated for each covariate, where the standardized difference was defined as the difference in means or proportions divided by the pooled standard deviation [2].

Using the IPTW method, HAE rates and safety outcomes were compared between the cohorts in the weighted sample. This included creating weighted estimates on rate ratios, mean difference and corresponding confidence intervals. For HAE rate, a weighted Poisson regression model was fitted to estimate adjusted rate ratios for the cohort variable.

## Supplementary tables

| Supplementary Table 1. Eligibility criteria for the systematic literature review | |
| --- | --- |
| Eligibility criteria | Details |
| Patients | Children (<18 years of age) with HAE type I or II^a^ or patients at any age with HAE with normal functioning C1-esterase inhibitor, as well as mixed population age group (type I, II and normal C1 inhibitor) |
| Intervention/  Comparators | Pharmacological only |
| Outcomes | Time of onset of symptom relief  Time to complete resolution of symptoms  The proportion of patients with a worsening intensity of HAE symptoms  Number of vomiting episodes  The proportion of patients receiving rescue study medication  Prophylaxis endpoints  Attack rate while on the study  The proportion of patients receiving rescue medication  Reduction in frequency of attacks  Breakthrough attacks while receiving prophylaxis  The severity of breakthrough attacks while receiving prophylaxis  Safety endpoints  Health-related quality of life endpoints |
| Study design | Randomized controlled trials  Prospective non-randomized interventional studies  Observational studies^b^ |
| Other | No language restrictions |
| Date^c^ | From database inception to October 20, 2022 |
| HAE: Hereditary angioedema; SLR: Systematic literature review.  Note: any study that focused on the mechanisms or activities of drugs on the body and was not directly related to the clinical efficacy of the treatment was excluded (i.e., maximum tolerated doses studies, dose-escalation studies, dose-limiting toxicity studies, pharmacokinetic/treatment mechanism studies).  ^a^Studies with a mixed population of adults and children with HAE type I or II were included.  ^b^Observational studies were not included in the initial search conducted in December 2016.  ^c^Original SLR was run from database inception to November 30, 2016, and final update was run on October 20, 2022. | |

| Supplementary Table 2. Baseline and prognostic factors of the SPRING and C1-INH studies as estimated per individual patient data | | | | |
| --- | --- | --- | --- | --- |
|  | Before IPTW | | After IPTW | |
| Regimen | SPRING trial cohort [3] (N=17) | C1-INH cohort [4] (N=12) | SPRING trial cohort [3] (N=17) | C1-INH cohort [4] (N=12) |
| Age, mean (SD), years | 8.29 (1.26) | 9.75 (1.29) | 8.66 (1.16) | 9.08 (1.36) |
| Sex, n (%) |  |  |  |  |
| Female | 10 (58.8) | 7 (58.3) | 69.3% | 67.4% |
| Male | 7 (41.2) | 5 (41.7) | 30.7% | 32.6% |
| Race, n (%) |  |  |  |  |
| Other | 1 (5.9) | 1 (8.3) | 7.6% | 8.9% |
| White | 16 (94.1) | 11 (91.7) | 92.4% | 91.1% |
| BMI, mean (SD), kg/m^2^ | 18.89 (4.44) | 19.36 (4.82) | 18.57 (4.00) | 18.41 (4.08) |
| Baseline attack rate, attacks/month, mean (SD) | 1.84 (1.65) | 3.43 (2.90) | 2.84 (2.50) | 3.05 (2.22) |
| BMI: Body mass index; HAE: Hereditary angioedema; ITC: Indirect treatment comparison; IPTW: Inverse probability of treatment weighting; SD: Standard deviation. | | | | |

| Supplementary Table 3. Baseline and prognostic factors considered for ITC analyses SPRING vs. Aygören Pürsün before and after IPTW | | | | |
| --- | --- | --- | --- | --- |
| Category/Statistic | SPRING trial cohort (N = 17) n (%) | C1-INH cohort  (N = 12) n (%) | Standardized difference | p-value |
| Baseline HAE attack rate before IPTW | | | | |
| Mean (SD) | 1.84 (1.65) | 3.43 (2.90) | −0.67 | 0.106 |
| Baseline HAE attack rate after IPTW | | | | |
| Mean (SD) | 2.46 (2.18) | 2.62 (2.26) | −0.07 | 0.854 |
| BMI: Body mass index; HAE: Hereditary angioedema; IPTW: Inverse probability of treatment weighting; ITC: Indirect treatment comparison; SD: Standard deviation. | | | | |

| Supplementary Table 4. Trial design of the SPRING and C1-INH 2019 studies used for indirect treatment comparison analysis | | |
| --- | --- | --- |
| Characteristic | SPRING trial cohort [3] | C1-INH study cohort [4] |
| Study design | Phase 3, open-label | Phase 3, single-blind, placebo-controlled |
| Trial type | Parallel | Crossover |
| Treatment groups | - Lanadelumab 150 mg Q4W - Lanadelumab 150 mg Q2W | - C1-INH 500 U and 1000 U twice weekly |
| Administration | - Subcutaneous injection | - Intravenous |
| Treatment duration | - 52 weeks | - 12 weeks before crossing over to the alternate dose for 12 weeks |
| Randomized population | - Confirmed HAE diagnosis (HAE type I/II) - Age 2 to < 12 years - History of HAE attacks: ≥1 investigator-confirmed HAE attack in the 3 months during baseline entered the 52-week lanadelumab treatment period | - Confirmed HAE diagnosis (HAE type I/II) - Age ≥6 to <12 years - Functional C1‐INH level <50% of normal - Average of ≥1.0 (≥2.0 in Germany) attacks/month of moderate or severe intensity or requiring acute treatment. |
| Primary endpoint | - Number of HAE attacks over the 52-week treatment period | - Monthly normalized number of attacks over the 12-week treatment period |
| C1-INH: C1-esterase inhibitor; HAE: Hereditary angioedema; Q2W: Every two weeks; Q4W: Every 4 weeks. | | |

**Supplementary Figure 1. Flow chart for the identification and selection of published sources for this indirect treatment comparison**. ITC: Indirect treatment comparison.


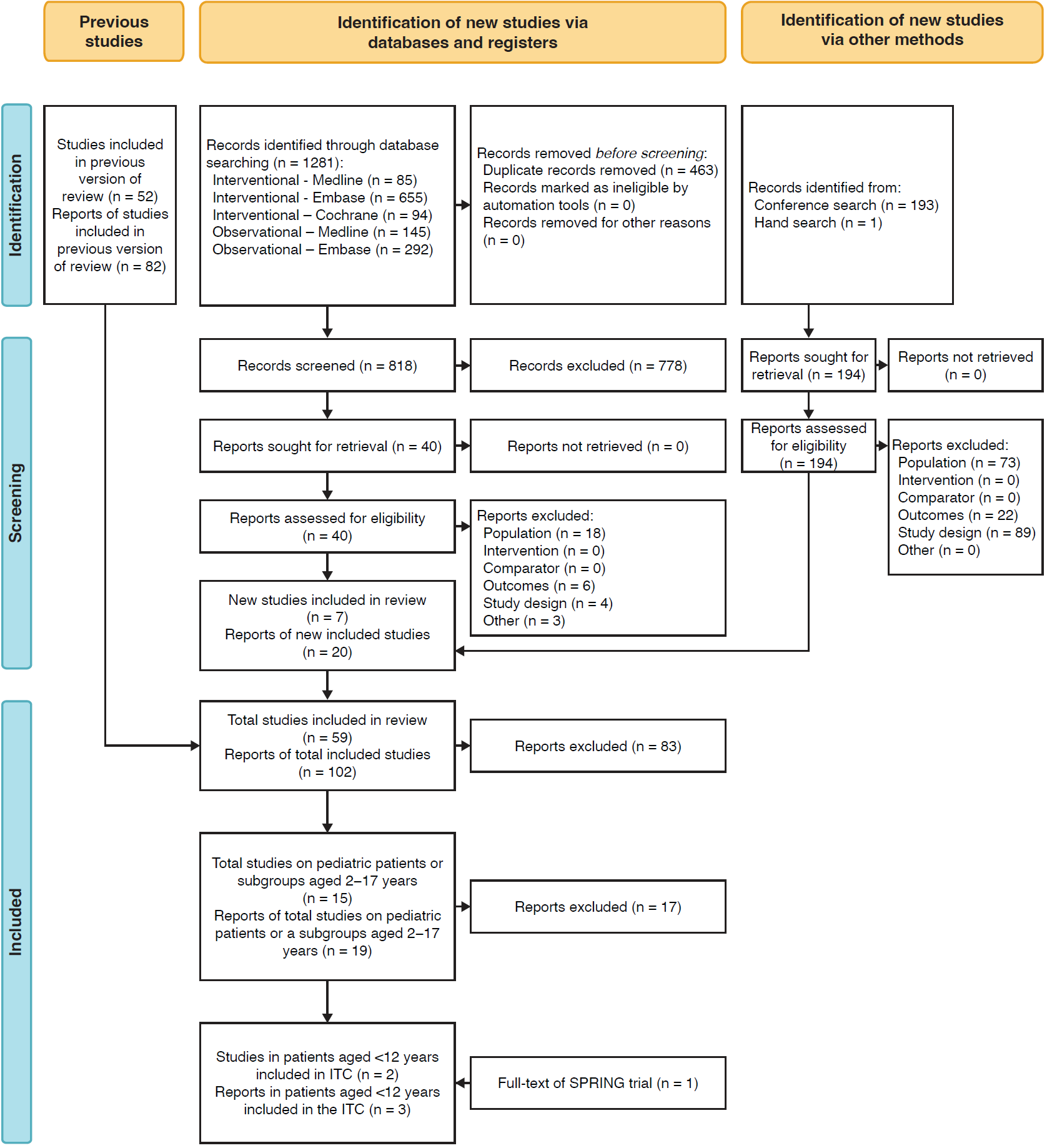


# References

1. Chesnaye NC, Stel VS, Tripepi G *et al.* An introduction to inverse probability of treatment weighting in observational research. *Clin Kidney J.* 15(1), 14-20 (2022).

2. Austin PC. An introduction to propensity score methods for reducing the effects of confounding in observational studies. *Multivariate Behav Res.* 46(3), 399-424 (2011).

3. Maurer M, Lumry WR, Li HH *et al.* Lanadelumab in patients 2 to less than 12 years old with hereditary angioedema: results from the phase 3 SPRING study. *J Allergy Clin Immunol Pract.* 12(1), 201-211.e6 (2024).

4. Aygören-Pürsün E, Soteres DF, Nieto-Martinez SA *et al.* A randomized trial of human C1 inhibitor prophylaxis in children with hereditary angioedema. *Pediatr Allergy Immunol.* 30(5), 553-561 (2019).
